# Feasibility trial of an integrated treatment “Activate for Life” for physical and mental well-being in older adults

**DOI:** 10.1093/geroni/igab046.3392

**Published:** 2021-12-17

**Authors:** Melba Hernandez-Tejada, Sundaravadivel Balasubramanian, Alexis Nagel, Mohan Madisetti, Teresa Kelechi

**Affiliations:** 1 University of Texas HSC at Houston, Houston, Texas, United States; 2 Medical University of South Carolina, Charleston, South Carolina, United States

## Abstract

This study describes the feasibility and patient satisfaction for an integrated treatment to address multiple health outcomes in a sample of older adults living in a low-income independent residence facility and their own homes in the community. Specifically, 30 older adults were offered the opportunity to participate in a feasibility study of different components of Activate for Life treatment targeting balance and physical strength (Otago Exercise Program), breathing retraining (Gentle Yoga and Yogic Breathing), and mental health (Behavioral Activation for Depression). Three treatment combinations were compared in a randomized repeated measures design to determine if adding components to the existing Otago program were feasible and if this affected patient satisfaction. Arm1: the Otago strength and balance program alone (n = 10); Arm 2: Otago + Gentle Yoga and Yogic Breathing (n = 10); and Arm 3: Otago + Gentle Yoga and Yogic Breathing + Behavioral Activation (we named this combination ‘Activate for Life’ n = 10). Dependent measures included recruitment rate, session completion characteristics, and satisfaction with the program. Overall, study and treatment components proved feasible, and participants reported high satisfaction with all 3 Arms.

